# Quantitative dual-energy CT for evaluating hepatocellular carcinoma after transarterial chemoembolization

**DOI:** 10.1038/s41598-021-90508-9

**Published:** 2021-05-27

**Authors:** Xiaofei Yue, Qiqi Jiang, Xuehan Hu, Chunyuan Cen, Songlin Song, Kun Qian, Yuting Lu, Ming Yang, Qian Li, Ping Han

**Affiliations:** 1 Department of Radiology, Union Hospital, Tongji Medical College, Huazhong University of Science and Technology, Wuhan, 430022 China; 2 Hubei Province Key Laboratory of Molecular Imaging, Wuhan, 430022 China; 3 Department of Nuclear Medicine, Union Hospital, Tongji Medical College, Huazhong University of Science and Technology, Wuhan, 430022 China

**Keywords:** Cancer imaging, Cancer, Liver cancer

## Abstract

We aimed to investigate the role of the quantitative parameters of dual-energy computed tomography (DECT) in evaluating patients with hepatocellular carcinoma (HCC) treated by transarterial chemoembolization (TACE). We retrospectively identified 80 HCC patients (mean age, 56 years; 61 men) treated by TACE who received contrast-enhanced DECT and were retreated by TACE within 7 days between November 2018 and December 2019. Taking digital subtraction angiography (DSA) and CT images as reference standard, two readers measured and calculated the values of normalized iodine concentration at arterial phase (NICAP), normalized iodine concentration at portal venous phase (NICPP), iodine concentration difference (ICD), arterial iodine fraction (AIF) and slope of the spectral Hounsfield unit curve (λ_Hu_) by placing matched regions of interests (ROIs) within the tumor active area (TAA), adjacent normal hepatic parenchyma (ANHP) and tumor necrotic area (TNA). Differences between the parameters were analyzed by the Kruskal–Wallis H test. Receiver operating characteristic analysis of the parameters performance in differentiating the three tissues types was performed. AIF exhibited a good performance in distinguishing TAA (0.93 ± 0.31) and ANHP (0.18 ± 0.14), the areas under the receiver operating characteristic curve (AUC) was 0.989, while the λ_Hu_ exhibited an excellent performance in distinguishing TAA (3.32 ± 1.24) and TNA (0.29 ± 0.27), with an AUC of 1.000. In conclusion, quantitative DECT can be effectively used to evaluate the tumor viability in HCC patients treated by TACE.

## Introduction

Hepatocellular carcinoma (HCC) is a common malignant tumor. The incipient symptoms of HCC are usually atypical^1^. Many patients are at Barcelona clinic liver cancer (BCLC) stage C at diagnosis^2^ and have lost the opportunity for curative surgery, resulting in a poor prognosis^3^. At present, the nonsurgical treatments of HCC consist mainly of radiofrequency ablation, microwave thermal ablation, transarterial chemoembolization (TACE), radiotherapy and systemic chemotherapy^4^.

TACE is the first-line treatment for the intermediate stage of HCC according to the BCLC system^5^. Using angiography, embolic agents and chemotherapeutic drugs are injected into the supply artery of the tumor, thereby reducing the blood supply and curbing disease progression^1^. However, TACE is complicated by postoperative recurrence^6^. Hence, imaging evaluation after TACE is very important.

The revised Response Evaluation Criteria in Solid Tumors (mRECIST) proposed in 2010^7^ and the Liver Imaging Reporting and Data System (LI-RADS) version 2018^8^ both set standards for the treatment response of liver cancer. The treatment response standards of both protocols are based on imaging features rather than a quantitative evaluation.

Conventional enhanced CT is an inexpensive, and convenient scan commonly used for post-TACE review of HCC. Nonetheless, the mixed energy image of a conventional CT scan may give rise to ray beam-hardening artifacts, and the high density of lipiodol deposition may cause a shielding effect on adjacent structures that affects the visual observation and evaluation.

Dual-energy computed tomography (DECT) provides a wide selection of dual energies by using stronger penetrating 150 Sn kV X-rays^9^. The efficiency of X-rays is improved by the “spectral filtering” effect, which contributes to a precise substance separation^10^. Iodine quantification is more accurate than standard enhancement measurements and DECT offers the measurement of iodine uptake rather than mere enhancement values^11^. Parameters of DECT such as slope of spectral Hounsfield unit curve (λ_Hu_) and arterial iodine fraction (AIF) are quantitative parameters that cannot be obtained with conventional CT^12–14^. Such measurements could effectively compensate for the lack of quantification on conventional CT.

The purpose of this study was to quantitatively differentiate the tumor active area (TAA), adjacent normal hepatic parenchyma (ANHP) and tumor necrotic area (TNA) after TACE using DECT parameters, thus providing an alternative method for the follow-up of patients with HCC and that may be used as a supplement to LI-RADS v2018.

## Methods

The protocol for this single-center study was approved by the Ethics Committee of Tongji Medical College of Huazhong University of Science and Technology and performed in accordance with the guidelines of the Declaration of Helsinki. All methods were performed in accordance with the relevant guidelines and regulations. All patients signed informed consent forms.

### Patient demographics

**Figure 1:**
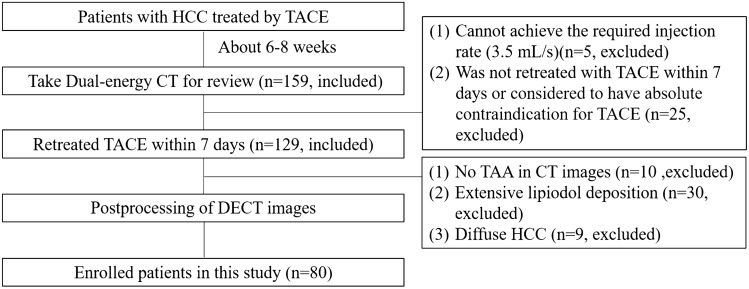
Patient enrollment workflow diagram.

Between November 2018 and December 2019, 80 consecutive patients (61men, 19 women) with HCC who underwent conventional TACE (C-TACE) (68 patients) or drug-eluting microsphere transarterial chemoembolization (DEM-TACE) (12 patients) intervention in the preceding 1–3 months were included in this study. Patients were enrolled according to the following criteria: (a) diagnosed with HCC^15^; (b) underwent TACE intervention in the preceding 1–3 months; (c) were to be retreated with TACE within 7 days after CT review. Among the 159 patients who were initially deemed qualified for the study, 79 were excluded for the following reasons: (a) could not achieve the required injection rate (3.5 mL/s, n = 5); (b) did not undergo retreatment with TACE within 7 days or were considered to have absolute contraindication for TACE^16^. (n = 25); (c) no TAA on CT images and DSA images(n = 10); (d) extensive lipiodol deposition, the area of non-lipiodol deposition regions was less than 0.5 cm^2^, leading to inaccurate measurement (n = 30); and (e) diffuse HCC with no ANHP to measure (n = 9). Figure 1 presents the patient enrollment workflow diagram.

### CT examinations

First, 1.2 mL/kg of iodine contrast medium (iodixanol, 320 mg I/mL; Hengrui Healthcare, Jiangsu, China) was intravenously injected. Then, third-generation dual-source CT (SOMATOM Force, Siemens Healthcare, Forchheim, Germany) was performed with multiple phases. Patients were instructed to fast for four hours before the CT scan.

All patients were positioned with their arms up on the scanning table in the supine position. Scanning was performed from the top of the diaphragm to the lower edge of the liver. First, an unenhanced CT scan of the abdomen was performed with a dual-energy model. Enhanced CT acquisitions were triggered using the bolus tracking technique, with the regions of interests (ROIs) placed at the beginning of the abdominal aorta. Arterial phase imaging was performed using automatic image-triggering software (preMonitoring; Siemens Healthcare). The arterial phase scan began after an 8-s delay at a rate of 3.5 mL/s, when the trigger attenuation threshold reached 100 HU. Then, 40 mL of saline solution was flushed at the same flow rate. A portal venous phase scan was conducted 24-s after the end of the arterial phase. The scanning protocol was as follows: collimator width, 128 × 0.6 mm; field of view (FOV), 300 mm; and pitch, 0.6. Tube A was operated at a peak voltage of 100 kV and 180 quality reference mAs (Siemens Healthcare). Tube B was operated at 150 Sn kV and 90 quality reference mAs. The data were reconstructed with a slice thickness of 1.5 mm.

### TACE retreatment

Digital subtraction angiography (DSA) examination is a part of TACE retreatment, and we only collected data from patients who required retreatment clinically without additional examinations. The Seldinger method is used to intubate the femoral artery via percutaneous puncture and insert the catheter into the abdominal trunk or common hepatic artery for DSA to acquire DSA images. Superior mesenteric artery angiography was performed, and attention was paid to finding collateral blood supply. The location, size, number and blood supply arteries of the tumor were determined by careful analysis of the angiographic findings. The chemotherapeutic drugs and embolic agents were mixed together and injected through the blood supply artery branch of the tumor^17^.

### Reference standard

Taking the DSA and CT images as references standard, two abdominal radiologists with more than five years of experience independently interpreted the CT images according to LI-RADS standards but were blinded to the DSA results. LI-RADS-Treatment Response (LR-TR) Viable was considered to be TAA with nodular, mass-like, or thick irregular tissue in or along the treated lesion with any of the following: arterial phase hyperenhancement; washout appearance; or enhancement similar to pretreatment. Two interventionalists with more than ten years of experience interpreted the TACE on picture archiving and communication system (PACS) and were blinded to the CT results; TAA was considered to exist when tumor staining was observed. A senior radiologist was consulted for any disagreement. Vital tissue was considered to exist when both the TACE and CT results suggested TAA.

### Postprocessing of DECT images

Images were transferred to a postprocessing software workstation (Syngo via, Siemens Healthcare). When there were multiple lesions, the three largest lesions were selected for measurement. Two types of reconstructed images were measured: color-coded iodine maps and monoenergetic images of 151 energies ranging from 40 to 190 keV. All ROIs of TAA, ANHP, TNA and abdominal aorta were manually plotted at the same location and size on arterial phase and portal venous phase to obtain the values of iodine concentration (IC) and standardized iodine concentration (NIC) on the iodine map. Spectral Hounsfield unit curves were obtained from the monoenergetic images of arterial phase. The normalized iodine concentration at arterial phase (NICAP), normalized iodine concentration at portal venous phase (NICPP), iodine concentration difference (ICD), arterial iodine fraction (AIF) and the slope of the spectral Hounsfield unit curve (λ_Hu_) were then calculated (Table 1).

**Table 1:** Calculation formula of each parameter.

Parameters	Full name	Formula
NICAP	Normalized iodine concentration at arterial phase	IClesion/ICaorta at arterial phase
NICPP	Normalized iodine concentration at portal venous phase	IClesion/ICaorta at portal venous phase
ICD	Iodine concentration difference	IC arterial phase − IC venous phase
AIF	Arterial iodine fraction	IC arterial phase/IC venous phase
λ_Hu_	The slope of the spectral Hounsfield unit curve	(HU_40kev_ − HU_70kev_)/30) at arterial phase

The λ_Hu_ was calculated as: λ_Hu_ = (HU_40kev_ − HU_70kev_)/30)^18,19^ on arterial phase. The HU_40keV_ indicates that the CT value was gauged on 40 keV images, and HU_70keV_ indicates that the CT value was gauged on 70 keV images. The NIC was calculated as: NIC = IC_lesion_/IC_aorta_, where IC_lesion_ and IC_aorta_ are the iodine level of the lesion and the aorta on the same slice, respectively; the iodine concentration in the lesion is normalized to the aortic iodine concentration to minimize the difference between patients^20^.

The ROIs of TAA, NAHP and TNA were round and were selected to avoid large blood vessels, iodized oil deposits and surrounding artifacts. For TNA, the area was selected in TACE-treated tumors that were not enhanced after repeated measurements by radiologists in three-phase enhanced images and single-energy images. ANHP was chosen to be close to the tumor and 1 cm away from it to avoid possible effects of microvascular invasion on the measured iodine concentration^17,21^. When measuring nodular lesions, tumor tissues were included as much as possible. When measuring massive lesions, multiple points were measured, and the mean values were calculated. All of the ROIs of one person were of the same size and at the same level of the scan, which depended on whichever had the smaller ROI. All data were measured at different times by two independent radiologists to avoid measurement errors, and the results were averaged.

### Statistical analyses

Continuous variables are reported as the mean ± standard deviation. All statistical analyses were performed with statistical software (SPSS, version 21.0, IBM Corporation, Armonk, NY, USA; MedCalc, version 15.2.2, MedCalc, Mariakerke, Belgium). The Kolmogorov–Smirnov test was used to test for normality. Consistency between the two radiologists was assessed by using ICC. The Kruskal–Wallis H test was used to compare the differences between various TAA, ANHP and TNA parameters. A value of *P* < 0.05 was considered significant. The diagnostic performance of each parameter (TAA, ANHP and TNA) was compared by plotting the ROC curve.

## Results

Relevant demographic and regions of interest (ROIs) characteristics were outlined in Table 2. The mean age at interview was 56 years, ranging from 24 to 78 years, A total of 80 patients (61 men and 19 women) were included in this study. A total of 445 ROIs were measured (194 in the TAA,194 in the ANHP and 57 in the TNA). The ROIs ranged from 0.5 to 6.3 cm^2^. with an average of 1.42 ± 0.92 cm^2^. A total of 23 patients had single lesions, while 57 patients had multiple lesions.

**Table 2:** Patients and lesions characteristics on DECT.

Characteristics	N
**Sex**	
Men	61
Women	19
Age (y)	56 ± 11
**Etiology of HCC**	
Hepatitis B virus	60
Alcoholism	1
Hepatitis C virus	6
Bilharziasis	3
Cryptogenic	14
**No. of HCC lesions per patient**	
Single	23
Multiple	57
**No. of ROIs**	
ROIs of the TAA	194
ROIs of the ANHP	194
ROIs of the TNA	57

The data are represented as the mean ± standard deviation or number of patients. HCC**,** hepatocellular carcinoma; ROIs, regions of interests; TAA, tumor active area; ANHP, adjacent normal hepatic parenchyma; TNA, tumor necrotic area.

### Intraclass correlation coefficients between two radiologists

The intraclass correlation coefficients (ICC) of the TAA, ANHP and TNA measurements were evaluated. The agreements of the measured values were good (between 0.760 and 0.977). The results are shown in Table 3.

**Table 3:** ICC of the measurements between two radiologists.

Groups	Parameters	ICC	95%CI
TAA	ICAP	0.968	0.958–0.976
	NICAP	0.977	0.969–0.982
	ICPP	0.977	0.969–0.982
	NICPP	0.975	0.967–0.981
ANHP	ICAP	0.874	0.830–0.908
	NICAP	0.863	0.815–0.899
	ICPP	0.954	0.936–0.967
	NICPP	0.936	0.913–0.954
TNA	ICAP	0.813	0.703–0.885
	NICAP	0.760	0.627–0.850
	ICPP	0.933	0.890–0.959
	NICPP	0.940	0.900–0.964

ICAP, iodine concentration at arterial phase; ICPP, iodine concentration at portal venous phase; NICAP, normalized iodine concentration at arterial phase; NICPP, normalized iodine concentration at portal venous phase; TAA, tumor active area; ANHP, adjacent normal hepatic parenchyma; TNA, tumor necrotic area.

### Differences in dual-energy CT parameters between different tissues

**Figure 2:**
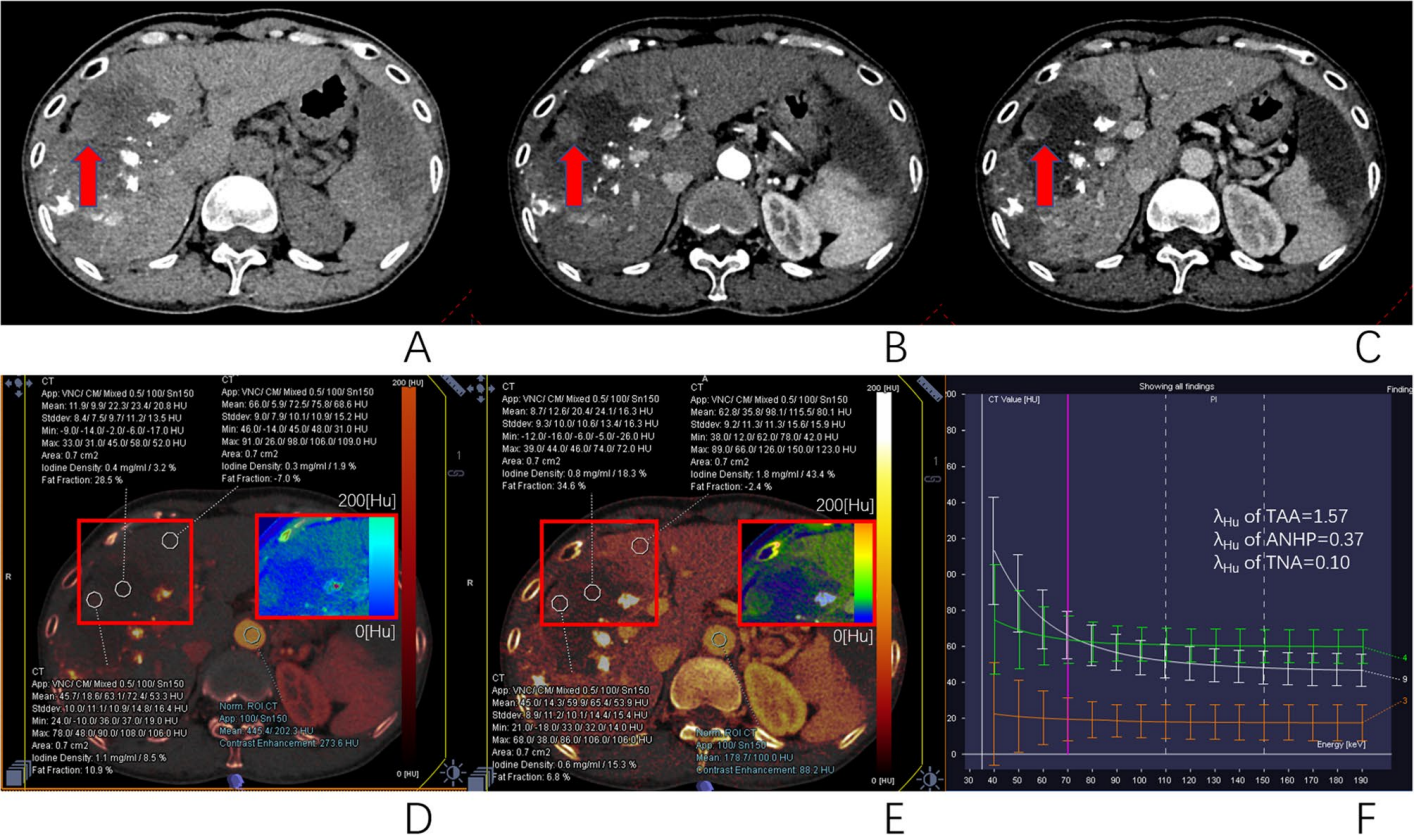
This is a patient who had a DECT examination 7 weeks after the conventional-TACE, showed that lipiodol deposited around the tumor, and TAA and TNA in the tumor after treatment. A large lesion with scattered lipiodol deposition is seen within the right lobe of the liver. The center non-enhancing area was TNA. Peripheral soft tissue nodularity is noted on axial non-enhanced CT (red arrow, **A**), with slight enhancement on arterial phase (**B**), and portal venous phase (**C**), which was considered by radiologists to be TAA. ROIs of TAA, ANHP, and TNA placed on arterial phase and portal phase iodine map (**D** and **E**) showed the IC and NIC of TAA to be 1.1 mg/ml and 8.5% at arterial phase, and 0.6 mg/ml and 15.3% at portal venous phase, respectively. The IC and NIC of ANHP was 0.3 mg/ml and 1.9% at arterial phase, and 1.8 mg/ml and 43.4% at portal venous phase, respectively. The IC and NIC of TNA was 0.4 mg/ml and 3.2% at arterial phase, and 0.8 mg/ml and 18.3% at portal venous phase, respectively. The λHu of TAA, ANHP and TNA were 1.57,0.37 and 0.10, respectively (**F**).

**Figure 3:**
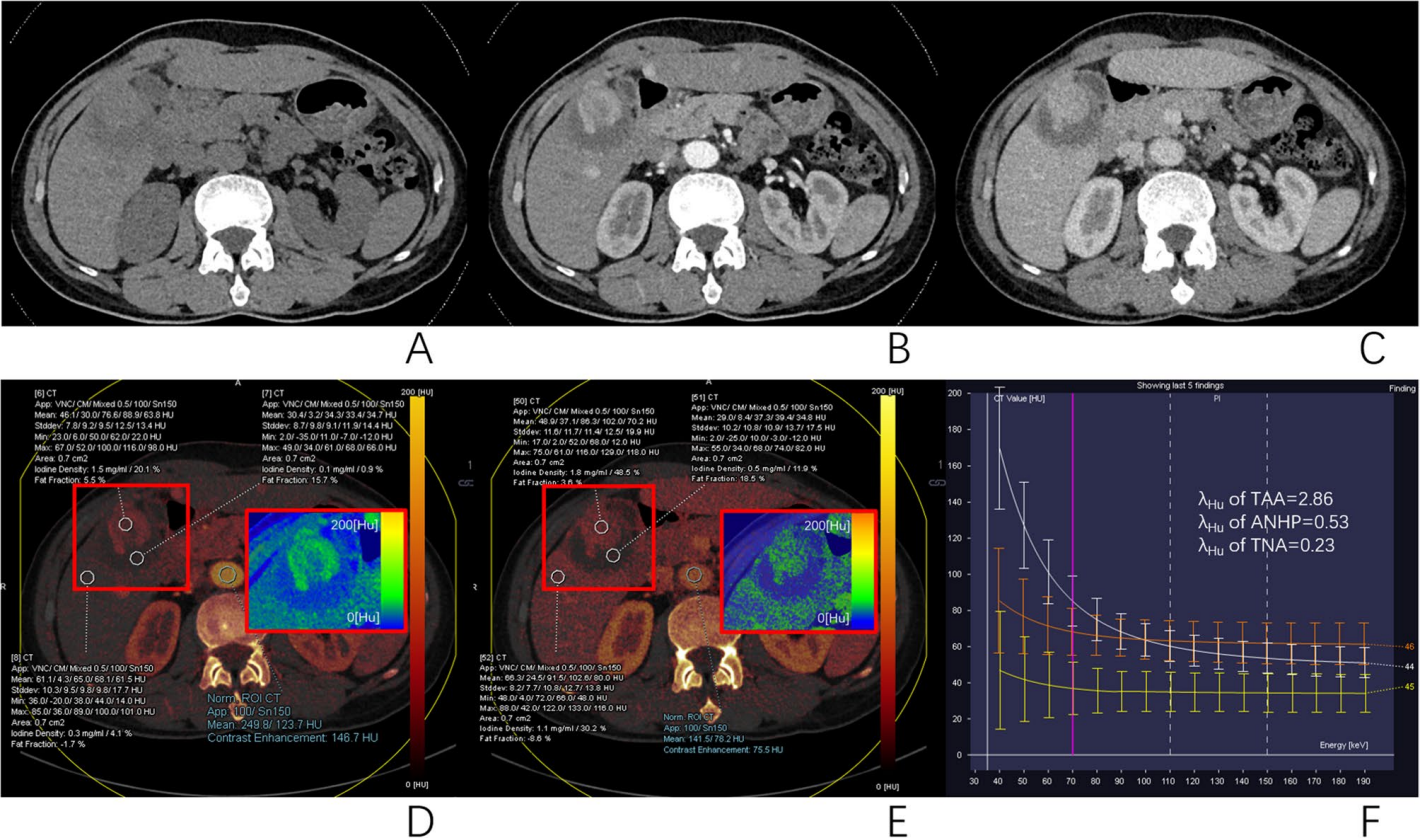
DECT images of a 58-year-old man with HCC undergoing DEM-TACE 6 weeks ago, showed that no lipiodol deposition in the original tumor lesions, but TAA and TNA were present. The right hepatic lobe showed a low-attenuation lesion on unenhanced CT image (**A**), significant enhancement at arterial phase (**B**), and persistent enhancement at the portal venous phase(**C**). TNA had no enhancement. ROIs of TAA, ANHP, and TNA placed on arterial and portal iodine map (**D** and **E**) showed IC and NIC of TAA to be 1.5 mg/ml and 20.1% at arterial phase and 1.8 mg/ml and 48.5% at portal venous phase, respectively. The IC and NIC of ANHP was 0.3 mg/ml and 4.1% at arterial phase, and 1.1 mg/ml and 30.2% at portal venous phase, respectively. The IC and NIC of TNA was 0.1 mg/ml and 0.9% at arterial phase and 0.5 mg/ml and 11.9% at portal venous phase, respectively. The λHu of TAA, ANHP and TNA were 2.86, 0.53 and 0.23, respectively (**F**).

**Figure 4:**
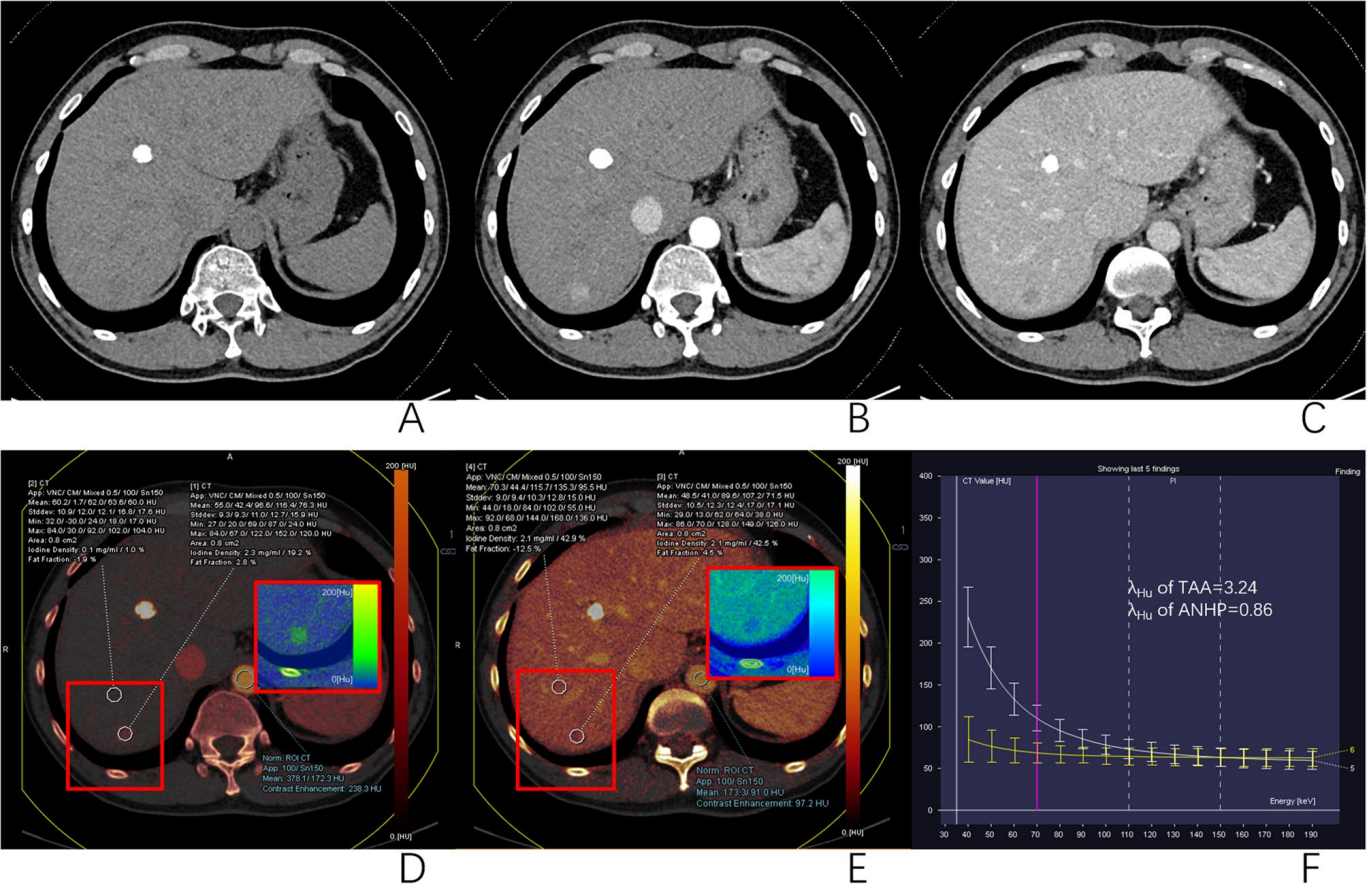
DECT images of a 52-year-old man with HCC undergoing lipiodol TACE. The right hepatic lobe showed the original lesions were completely deposited with lipiodol, and the new lesions appeared, a low-attenuation nodule on axial unenhanced CT image (**A**), significant enhancement at the arterial phase (**B**), and wash-out at the portal venous phase(**C**). ROIs of TAA and ANHP (**D** and **E**) placed on arterial and portal iodine map showed IC and NIC of TAA to be 2.3 mg/ml and 19.2% at arterial phase, and 2.1 mg/ml and 42.5% at portal venous phase. IC and NIC of ANHP was 0.1 mg/ml and 1.0% at arterial phase, and 2.1 mg/ml and 42.9% at portal venous phase. The λ_Hu_ of TAA and ANHP was 3.24 and 0.86, respectively (**F**).

The differences in parameters between different tissues are shown in Table 4. The λ_Hu_, normalized iodine concentration at arterial phase (NICAP) and normalized iodine concentration at portal venous phase (NICPP) of the TAA were 3.32 ± 1.24, 16.51 ± 6.75% and 46.68 ± 15.10%, respectively, which were significantly higher than those in the ANHP(0.71 ± 0.39, 2.66 ± 2.05% and 39.48 ± 10.65%, respectively, *P* < 0.05) and TNA (0.29 ± 0.27, 2.28 ± 1.47%, and 9.30 ± 7.46%, respectively, *P* < 0.05); The iodine concentration difference(ICD) of the TAA was 0.56 ± 0.46 mg/mL, which was lower than that of ANHP (1.49 ± 0.54 mg/mL). The AIF of the TAA was 0.93 ± 0.31, which was significantly higher than that of the ANHP (0.18 ± 0.14, *P* < 0.05) but not significantly different from the TNA (0.99 ± 0.86, *P* = 0.251). Figures 2, 3 and 4 provide representative images of the DECT parametric maps.

**Table 4:** Comparison of various parameters in different liver tissues after TACE.

Parameters	TAA (mean ± SD)	ANHP (mean ± SD)	TNA (mean ± SD)	*P*
λ_Hu_	3.32 ± 1.24*^&^	0.71 ± 0.39^#^	0.29 ± 0.27	< 0.05
NICAP (%)	16.51 ± 6.75*^&^	2.66 ± 2.05	2.28 ± 1.47	< 0.05
NICPP (%)	46.68 ± 15.10*^&^	39.48 ± 10.65^#^	9.30 ± 7.46	< 0.05
ICD(mg/mL)	0.56 ± 0.46*^&^	1.49 ± 0.54^#^	0.29 ± 0.36	< 0.05
AIF	0.93 ± 0.31*	0.18 ± 0.14^#^	0.99 ± 0.86	< 0.05

λ_Hu_, slope of the spectral Hounsfield unit curve; NICAP, normalized iodine concentration at arterial phase; NICPP, normalized iodine concentration at portal venous phase; ICD, iodine concentration difference; AIF, arterial iodine fraction; TAA, tumor active area; ANHP, adjacent normal hepatic parenchyma; TNA, tumor necrotic area.

*Indicates a significant difference between TAA and ANHP (*P* < 0.05).

^#^Indicates a significant difference between TNA and ANHP (*P* < 0.05).

^&^Indicates a significant difference between TAA and TNA (*P* < 0.05).

There were no significant differences in the parameters results of 12 patients treated by DEM-TACE and 68 patients treated by lipiodol TACE. The corresponding results were added in the supplementary materials.

### Diagnostic performance of the parameters across different tissues

**Figure 5:**
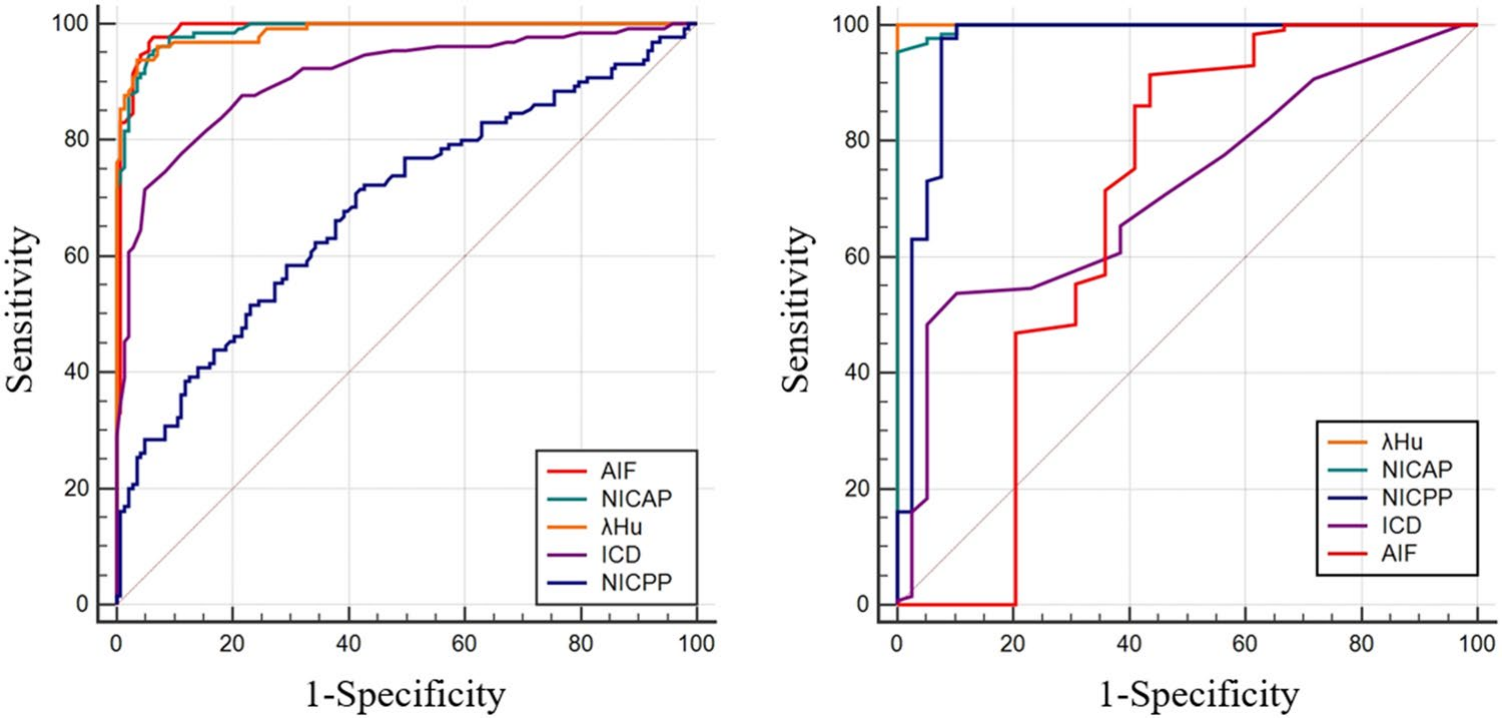
ROC curves of the AIF, ICD, NICAP, NICPP and λ_Hu_ performance in distinction of TAA and ANHP (**A**), and TAA and TNA (**B**). λ_Hu_, slope of the spectral Hounsfield unit curve; NICAP, normalized iodine concentration at arterial phase; NICPP, normalized iodine concentration at portal venous phase; ICD, iodine concentration difference; AIF, arterial iodine fraction.

The diagnostic performance of the parameters across different tissues is summarized in Table 5. The receiver operating characteristic (ROC) curve showed that λ_Hu_, NICAP, ICD, and AIF have a high efficiency for the differential diagnosis of the TAA and ANHP (areas under the ROC curve (AUC): 0.987, 0.988, 0.909, and 0.989, respectively). All of the parameters exhibited considerable sensitivity and specificity. The NICPP exhibited low efficiency for the discrimination of the TAA and ANHP (AUC 0.686). λ_Hu_, NICAP, and NICPP exhibited high performance for the differential diagnosis of the TAA and TNA (AUC: 1.000,0.997, and 0.962, respectively). The ICD and AIF exhibited weak performance for the differential diagnosis of the TAA and TNA (AUC: 0.717, and 0.688, respectively) (Fig. 5).

**Table 5:** Diagnostic performance of each parameter in distinction of different tissues.

	Parameter	AUC (95%CI)	Youden index	Sensitivity (%)	Specificity (%)	Threshold value
TAA and ANHP	λ_Hu_	0.987 (0.965–0.997)	0.92	95.88	96.50	1.37
	NICAP (%)	0.988 (0.967–0.997)	0.91	96.39	94.41	6.55
	NICPP (%)	0.686 (0.628–0.741)	0.27	68.04	58.74	40.15
	ICD	0.909 (0.868–0.940)	0.67	71.54	95.10	0.70
	AIF	0.989 (0.968–0.998)	0.93	96.91	95.80	0.47
TAA and TNA	λ_Hu_	1.000 (0.978–1.000)	0.98	99.48	98.25	0.85
	NICAP (%)	0.997 (0.973–1.000)	0.97	96.91	100.00	6.10
	NICPP (%)	0.962 (0.921–0.985)	0.92	99.48	92.98	16.9
	ICD	0.717 (0.643–0.784)	0.44	53.85	89.74	0.40
	AIF	0.688 (0.612–0.757)	0.33	94.33	38.60	0.50

λ_Hu_, Slope of the spectral Hounsfield unit curve; NICAP, normalized iodine concentration at arterial phase; NICPP, normalized iodine concentration at portal venous phase; ICD, iodine concentration difference; AIF, arterial iodine fraction; TAA, tumor active area; ANHP, adjacent normal hepatic parenchyma; TNA, tumor necrotic areas.

## Discussion

DECT has advantages in providing artifact reduction^22^, additional information regarding tissue composition^23,24^, and radiation dose reduction^25^. In this study, we found that the DECT parameters, especially λ_Hu_ at the arterial phase and NICAP, showed high efficiency for discriminating the different tissues in patients with HCC, with an AUC values of 0.987 and 0.988, for the recognition of the TAA and ANHP, respectively, and an AUC values of 1.000, and 0.997 for the recognition of the TAA and TNA, respectively. The quantitative parameters of DECT may help physicians to judge the effect of treatment and make correct clinical decisions.

The linear decay coefficient of tissues decreases with increasing keV values. However, the decline rate of each tissue is different^13,26^, that is, the λ_Hu_ can show energy attenuation and depends on the physical and chemical properties of the material itself. The CT value of the material is greatly attenuated when energy changes are maintained at a low level. The energy spectrum curve of the TAA measured in this study was steep and straight from 40_keV_ to 70_keV_, consistent with the literature^18,19^. The λ_Hu_ can significantly reflect the blood supply characteristics of different tissues at arterial phase. The λ_Hu_ of different tissues in this study showed a decline with varying slopes, among which the λ_Hu_ of the TAA was significantly larger than those of the ANHP and TNA. ROC analysis showed that the λ_Hu_ was highly effective in discriminating various tissues. A value higher than 1.32 was more likely to indicate a TAA. Therefore, the λ_Hu_ is of great value in the follow-up post TACE. Changes in the λ_Hu_ in colon cancer patients with varying degrees of differentiation have also been compared^27^. The results showed that poorly differentiated or undifferentiated carcinoma had a larger λ_Hu_ than well-differentiated carcinoma, providing a feasible basis for the evaluation of the tumor pathology. Therefore, evaluation of the degree of differentiation of tumors after TACE should be further studied.

Functional information regarding the microcirculation of the normal parenchyma and liver focal tumor lesions can be provided by CT perfusion imaging of the liver, which is a promising technology for the diagnosis of metastatic or primary tumors and for the evaluation of the efficacy of tumor treatment. CT perfusion imaging clinical application has not been widely promoted because of the large radiation dose. Mule S, et al. have shown arterial IC to be significantly related to both arterial blood flow and blood volume, confirming the ability of DECT to evaluate both morphological and perfusion changes^28^. Sonja Gordic, et al. found strong intra-individual correlations between iodine density and arterial perfusion (*r* = 0.75)^29^. Our study showed that NICAP and NICPP had high sensitivity and specificity in distinguishing TAA from TNA. NICAP had high sensitivity and specificity in distinguishing TAA from ANHP, which indicates that NICAP and NICPP could reflect the perfusion information of liver tissue and distinguish TAA from TNA and ANHP in the liver.

The current standard for treatment response after TACE is based on morphology, which depends on the experience of the radiologist, DECT parameters could provide quantitative information as a supplement to mRECIST and LI-RADS. Especially for atypical tumors, the quantitative parameters of DECT may help physicians to better judge the effect of treatment and make correct clinical decisions. However quantitative analysis has not been widely promoted, mostly due to the lack of uniformity of equipment and parameters, and some studies are single-center studies, making it difficult to generalize the research results.

The application of DECT in abdominal imaging has been proved to be beneficial, and our research confirms its usefulness compared with compared to conventional CT exam performed at 120 kV, the radiation dose is not increased with DECT and may even be reduced, which benefits from optimization of solutions such as iterative reconstruction technology and the tube current modulation. Thus, the wide application of DECT will not result in an additional radiation dose burden to patients^30^.

In our study, lipiodol TACE and DEM-TACE treatments were included. There were no significant differences between the two methods in the parameters of this study. The relevant content was discussed in the supplementary materials.

Our study had some limitations. First, the number of patients was small, and this was a single-center study. If additional data can be obtained, large-scale multicenter studies may more realistically and accurately confirm the results. Second, the pathological results of some patients were not obtained, because patients treated with TACE rarely undergo needle biopsy or surgical resection.

In conclusion, DECT can effectively and quantitatively distinguish different HCC tissues after TACE and may be considered supplementary to LI-RADS v2018 for assessing HCC patients post TACE.

## Supplementary Information

Supplementary Information 1.

## Abbreviations

HCC: Hepatocellular carcinoma
TACE: Transarterial chemoembolization
NICAP: Normalized iodine concentration at arterial phase
NICPP: Normalized iodine concentration at portal venous phase
λ_Hu_: Slope of spectral Hounsfield unit curve
ICD: Iodine concentration difference
AIF: Arterial iodine fraction
TAA: Tumor active area
ANHP: Adjacent normal hepatic parenchyma
TNA: Tumor necrotic area
