# Supplementary material for: Quantitative dual-energy CT for evaluating hepatocellular carcinoma after transarterial chemoembolization
Source: Sci Rep. 2021 May 27;11:11127. doi: 10.1038/s41598-021-90508-9 (PMC8160271; doi:10.1038/s41598-021-90508-9)
Supplement: Supplementary file 1 — Supplementary Information 1. [file 41598_2021_90508_MOESM1_ESM.docx]

**Supplemental Material**

**Quantitative Dual-Energy CT for Evaluating Hepatocellular Carcinoma after Transarterial Chemoembolization**

Xiaofei Yue^1,2,+^, Qiqi Jiang^1,2,+^, Xuehan Hu^2,3^, Chunyuan Cen^1,2^, Songlin Song^1,2^, Kun Qian^1,2^, Yuting Lu^1,2^, Ming Yang^1,2^, Qian Li^1,2^, Ping Han*^1,2^

1.Department of Radiology, Union Hospital, Tongji Medical College, Huazhong University of Science and Technology, Wuhan, 430022, China;

2.Hubei Province Key Laboratory of Molecular Imaging, Wuhan, 430022, China;

3.Department of Nuclear Medicine, Union Hospital, Tongji Medical College, Huazhong University of Science and Technology, Wuhan 430022, China.

^+^ Xiaofei Yue and Qiqi Jiang contributed equally to this work

First author: Xiaofei Yue, Email: erinyue8348@126.com, Tel: +86-131-6338-0813

Co-first author: Qiqi Jiang, Email: 296943129@qq.com, Tel: +86-156-2378-0779

*Corresponding author:

Ping Han, M.D., Ph.D.

Department of Radiology, Union Hospital, Tongji Medical College, Huazhong University of Science and Technology.

No. 1277 Jiefang Avenue, Wuhan, Hubei, 430022, China.

E-mail: hanping_uh@hust.edu.cn

Tel: +86-137-0717-0023

Fax: +86-27-85726020

**Supplemental Results:**

| **Parameters** | **TAA (mean ± SD)** | | |  | **ANHP (mean ± SD)** | | |  | **TNA (mean ± SD)** | | |
| --- | --- | --- | --- | --- | --- | --- | --- | --- | --- | --- | --- |
|  | lipiodol TACE | DEM-TACE | P |  | lipiodol TACE | DEM-TACE | P |  | lipiodol TACE | DEM-TACE | P |
| λ_Hu_ | 3.34±1.26 | 3.14±1.11 | 0.31 |  | 0.73±0.39 | 0.62±0.38 | 0.19 |  | 0.29±0.29 | 0.31±0.12 | 0.17 |
| NICAP (%) | 16.12±6.44 | 18.26±8.28 | 0.06 |  | 2.59±2.01 | 3.13±2.29 | 0.84 |  | 2.32±1.54 | 2.07±1.09 | 0.92 |
| NICPP (%) | 46.44±15.10 | 48.24±15.40 | 0.48 |  | 39.87±10.99 | 36.56±7.32 | 0.25 |  | 9.87±7.77 | 6.62±5.22 | 0.10 |
| ICD (mg/mL) | 0.55±0.47 | 0.38±0.32 | 0.14 |  | 1.54±0.55 | 1.44±0.58 | 0.26 |  | 0.24±0.34 | 0.18±0.22 | 0.85 |
| AIF | 0.92±0.30 | 1.05±0.31 | 0.24 |  | 0.18±0.13 | 0.22±0.17 | 0.52 |  | 0.99±0.88 | 0.95±0.77 | 0.99 |

**Supplemental Table 1.** Results of the parameters comparison between DEM-TACE and lipiodol TACE. λ_Hu_, slope of the spectral Hounsfield unit curve; NICAP, normalized iodine concentration at arterial phase; NICPP, normalized iodine concentration at portal venous phase; ICD, iodine concentration difference; AIF, arterial iodine fraction; TAA, tumor active area; ANHP, adjacent normal hepatic parenchyma; TNA, tumor necrotic area. DEM-TACE, drug-eluting microsphere transarterial chemoembolization.

**Supplemental Discussion:**

TACE and transarterial embolization(TAE) are two widely used locoregional therapies for HCC, especially for intermediate and advanced HCCs^1^. TACE treatment is divided into conventional TACE(C-TACE) and drug-eluting beans TACE (DEB-TACE). The study shows that DEB-TACE has a better overall survival rate and response to HCC than C-TACE^2^, but the incidence of local complications related to surgery is higher than that of C-TACE^3,4^. At present, the data are not sufficient to prove that DEB-TACE is superior to C-TACE in efficacy and safety^5^, but the findings do indicate that DEB-TACE can be used as an alternative and equivalent treatment method for C-TACE^6,7^. The transcatherial embolization (TAE) is used to block the blood supply artery of liver tumor with embolization alone. The therapeutic effect of TAE is not significantly different from that of TACE^8,9^, and its use is still recommended ^10^.

**Supplemental References**

1 Lanza, E. *et al.* Transarterial Therapies for Hepatocellular Carcinoma. *Liver Cancer* **6**, 27-33, doi:10.1159/000449347 (2016).

2 Ni, J. Y., Xu, L. F., Wang, W. D., Sun, H. L. & Chen, Y. T. Conventional transarterial chemoembolization vs microsphere embolization in hepatocellular carcinoma: a meta-analysis. *World J Gastroenterol* **20**, 17206-17217, doi:10.3748/wjg.v20.i45.17206 (2014).

3 Ma, Y. *et al.* Comparison of treatment efficacy and safety between drug-eluting bead transarterial chemoembolization with CalliSpheres((R)) microspheres and conventional transarterial chemoembolization as first-line treatment in hepatocellular carcinoma patients. *Am J Transl Res* **11**, 7456-7470 (2019).

4 Monier, A. *et al.* Liver and biliary damages following transarterial chemoembolization of hepatocellular carcinoma: comparison between drug-eluting beads and lipiodol emulsion. *Eur Radiol* **27**, 1431-1439, doi:10.1007/s00330-016-4488-y (2017).

5 Chang, Y., Jeong, S. W., Young Jang, J. & Jae Kim, Y. Recent Updates of Transarterial Chemoembolilzation in Hepatocellular Carcinoma. *Int J Mol Sci* **21**, doi:10.3390/ijms21218165 (2020).

6 Boily, G. *et al.* Transarterial embolization therapies for the treatment of hepatocellular carcinoma: CEPO review and clinical recommendations. *HPB (Oxford)* **17**, 52-65, doi:10.1111/hpb.12273 (2015).

7 Golfieri, R. *et al.* Randomised controlled trial of doxorubicin-eluting beads vs conventional chemoembolisation for hepatocellular carcinoma. *Br J Cancer* **111**, 255-264, doi:10.1038/bjc.2014.199 (2014).

8 Lee, E. W. & Khan, S. Recent advances in transarterial embolotherapies in the treatment of hepatocellular carcinoma. *Clin Mol Hepatol* **23**, 265-272, doi:10.3350/cmh.2017.0111 (2017).

9 Brown, K. T. *et al.* Randomized Trial of Hepatic Artery Embolization for Hepatocellular Carcinoma Using Doxorubicin-Eluting Microspheres Compared With Embolization With Microspheres Alone. *J Clin Oncol* **34**, 2046-2053, doi:10.1200/JCO.2015.64.0821 (2016).

10 Makary, M. S., Khandpur, U., Cloyd, J. M., Mumtaz, K. & Dowell, J. D. Locoregional Therapy Approaches for Hepatocellular Carcinoma: Recent Advances and Management Strategies. *Cancers (Basel)* **12**, doi:10.3390/cancers12071914 (2020).
